# Effect of manual therapy with exercise in patients with chronic cervical radiculopathy: a randomized clinical trial

**DOI:** 10.1186/s13063-021-05690-y

**Published:** 2021-10-18

**Authors:** Ali M. Alshami, Duaa A. Bamhair

**Affiliations:** 1 Department of Physical Therapy, Imam Abdulrahman Bin Faisal University, Dammam, Saudi Arabia; 2 Department of Physical Therapy, East Jeddah Hospital, Jeddah, Saudi Arabia

**Keywords:** Neck pain, Pain sensitivity, Pain threshold, Physical therapy, Spinal manipulation

## Abstract

**Background:**

Research that has examined the effects of cervical spine mobilization on hypoesthesia and hypersensitivity characteristics in patients with cervical radiculopathy is scarce. The aim of this study was to examine the short-term effects of vertebral mobilization on the sensory features in patients with cervical radiculopathy.

**Methods:**

Twenty-eight participants with chronic cervical radiculopathy were randomly allocated to (1) an experimental group [cervical vertebral mobilization technique and exercise] or (2) a comparison group [minimal superficial circular pressure on the skin and exercise]. Participants received a total of 6 sessions for 3–5 weeks. Numeric Pain Rating Scale (NPRS), Neck Disability Index (NDI), pressure pain threshold (PPT), heat/cold pain threshold (HPT/CPT), and active cervical range of motion (ROM) were measured at baseline immediately after the first session and after the sixth session.

**Results:**

The experimental group showed improvements from baseline to session 6 in NPRS [mean difference 2.6; 95% confidence interval: −4.6, −0.7], NDI [14; −23.3, −4.3], and active cervical ROM in extension [14°; 2.3, 25.5], rotation [16°; 8.8, 22.5], and lateral flexion to the affected side [10°; 2.3, 16.8]. Improvements were also found in PPT at the neck [124 kPa; 57, 191.1] and C7 level at the hand [99 kPa; 3.6, 194.9]. There were no changes in the HPT and CPT at any tested area (*P*>0.050).

**Conclusions:**

Cervical vertebral mobilization for patients with chronic cervical radiculopathy reduced localized mechanical, but not thermal, pain hypersensitivity.

**Trial registration:**

ClinicalTrials.gov (NCT03328351). Registered on November 1, 2017, retrospectively registered.

**Supplementary Information:**

The online version contains supplementary material available at 10.1186/s13063-021-05690-y.

## Background

Cervical radiculopathy is a relatively common disorder that is characterized by dysfunction of the spinal nerve or the nerve roots due to mechanical compression or inflammation. Epidemiologic data on cervical radiculopathy are sparse [1]. The most widely cited study is a large population-based study in Rochester, USA, by Radhakrishnan et al. (1994) who found that the average annual incidence of cervical radiculopathy between 1976 and 1990 per 100,000 people was 83.2. In a more recent study from the US military, the incidence of cervical radiculopathy was 1.79 per 1000 person-years [2]. In Saudi Arabia, the prevalence of cervical radiculopathy among patients who were diagnosed with neck disorders was estimated to be 4.2% between 2011 and 2013 [3].

The etiology of cervical radiculopathy is commonly attributed to mechanical compression or chemical irritation of the cervical nerve roots [4]. The most contributing factor is probably related to foraminal stenosis due to osteoarthritic changes within the joints of the cervical spine, rather than disc herniation [5]. Disc degenerative disease decreases foraminal height and results in osteophyte formation. Other problems that decrease the intervertebral foramen include trauma, infection, and tumor [4].

Symptoms of cervical radiculopathy may include neck and upper limb pain as well as neurological signs such as muscle weakness, disturbed sensation, and decreased reflex [6]. Patients with cervical radiculopathy demonstrated characteristics of sensory changes such as hypoesthesia (mechanical, thermal, and vibratory) and cold and pressure pain hypersensitivity [7, 8] in the most painful area and symptomatic dermatome [8–10].These sensory changes were measured by quantitative sensory tests (QSTs) such as pressure pain threshold (PPT), cold pain threshold (CPT), and heat/cold detection threshold (HDT/CDT).

Conservative treatment, particularly physical therapy, is generally recommended as initial treatment for patients with cervical radiculopathy [11, 12]. Physical therapy includes, but is not limited to, therapeutic exercises [13], mechanical and manual cervical traction [11, 14], and cervical collar [12]. Previous studies also found that vertebral mobilization and manipulation of the cervical spine were effective in improving pain, neck movement, and function in patients with cervical radiculopathy [15–19]. These studies investigated the effect of vertebral mobilization using outcome measures such visual analog scale (VAS), neck movement, strength of neck muscles, neck disability index (NDI), and 36-short form health survey (SF-36). Studies that investigated the effects of cervical mobilization on the sensory features by measuring QSTs in patients with cervical radiculopathy are lacking. Therefore, this RCT aimed to compare a treatment program that included cervical vertebral mobilization to a treatment program that included minimal superficial pressure on sensory changes in patients with chronic cervical radiculopathy. We tested the hypothesis that sensory features in patients with cervical radiculopathy can be changed by cervical vertebral mobilization techniques.

## Methods

### Study design and setting

This double-blind randomized clinical trial was registered with ClinicalTrials.gov (NCT 03328351) and followed the guidelines of Consolidated Standards of Reporting Trials (CONSORT). Ethical approvals were obtained from the Institutional Review Board at the University of Dammam, Saudi Arabia (IRB-PGS-2016-03-142, date: October 2, 2016) and the Saudi Ministry of Health (A00395, date: December 18, 2016) before initiation. The procedures followed during the study were in accordance with the Helsinki Declaration. Patients were blind and not fully informed about the study purpose and interventions. The assessor (an independent therapist) who performed the outcome measurements was blind to the participants’ groups. The study was carried out at the outpatient departments of physical therapy in King Abdul-Aziz Hospital and East Jeddah General Hospital in Jeddah, Saudi Arabia, from December 2016 to August 2017.

### Sample size calculation

The sample size was calculated using G*Power program version 3.1.9.2 (Heinrich-Heine-University Dusseldorf, Germany). The results of PPT on the neck of 10 participants (experimental group *N* = 5, mean PPT 491.9 kPa; comparison group *N* = 5, mean PPT 306.6 kPa) were used to calculate the sample size. The following combination was used for sample calculation: a priori power analysis of variance (ANOVA), repeated measures, within-between interaction, alpha level of 0.05, power (1-*β*) of 80%, with 2 groups and 3 measurements, non-sphericity correction (*Є*) of 1, and an effect size (*f*) of 0.446. The effect size was calculated by G*Power using partial eta squared (*η*^2^) of 0.166 of the difference between mean 1 and mean 2 that was resulted from ANOVA table. The required sample size was estimated at 14 participants per group.

### Participants

A sample of convenience was applied to recruit adult patients with ≥ 3 months history of neck pain that radiated to only one upper extremity (unilateral) with one or more level of nerve root involvement. Participants were classified as having cervical radiculopathy based on the presence of nerve root dysfunction features (weak myotome or diminished/absent reflex and diminished/absent sensation) [20] as well as the presence of ≥ 3 criteria of Wainner et al. [21] (Spurling test, distraction test, upper limb neurodynamic test 1, and cervical rotation towards painful side < 60°). Participants were excluded if they had osteoporosis, tumor, metabolic disease, resting blood pressure ≥ 149/90 mmHg [22], rheumatoid arthritis, whiplash injury, cervical myelopathy, pregnancy [17, 23], past surgery to the cervical or thoracic spine [23], neurological disorders, diabetes [24], or were unable to read and speak Arabic or English.

The treating therapist, who was a physical therapist with more than 5 years of clinical experience, assessed participants for eligibility and then assessed the cervical spine to identify the target level for treatment. The treating therapist applied posterior anterior (PA) glide centrally on the cervical spinous process and unilaterally on the articular pillars to determine the most painful level. The supposed affected dermatome was established by testing skin sensation with a pin prick as described in a previous protocol [20]. Once a participant was deemed eligible and accepted participation in the study, they provided a written consent form (Additional file 1).

### Randomization

The assessor, a physical therapist with more than 5 years of clinical experience, generated a random allocation sequence using Graphpad software (https://www.graphpad.com/) before the initiation of the study. Twenty-eight numbers were uniquely randomized in equal number to two different groups (14 numbers for experimental group and 14 numbers for comparison group). Each number with its allocated group was written on a piece of paper and concealed in an envelope. The participants were asked to select an envelope to be randomly allocated to one of the groups in a parallel design (1:1 ratio): experimental group or comparison group.

### Interventions

The treating therapist provided all interventions. Before the first session started, all participants in both groups were provided with a standardized verbal education about pain based on a previous protocol [25, 26]. The experimental group received an individualized cervical mobilization technique and exercise, whereas the comparison group received minimal superficial pressure on the skin and exercise (Additional file 2). Each participant had 6 treatment sessions over 3–5 weeks, similar to a previous protocol [16].

### Experimental group

The following cervical vertebral mobilization technique was used based on the participants’ responses (i.e., reduction and/or centralization of symptoms): posterior-anterior (PA) or lateral vertebral glides. For the PA vertebral glides, the participant was in a prone position and the treating therapist stood at the side of the participant’s head. The tips of the thumbs were placed in opposition at the level of the spinous process (for central PA) or at the level of the facet joint (for unilateral PA) of the cervical vertebra. Then, the treating therapist applied an oscillatory pressure of grade three on the most painful level [17, 27] for 2 min and 3 sets [28]. For the cervical lateral vertebral glides, the participant was supine, and the treating therapist placed the symptomatic patient’s upper limb into an upper limb neurodynamic test 1 (median nerve bias) position as tolerated: abduction of the shoulder, lateral rotation of the shoulder, supination of the forearm, extension of the wrist and finger, and extension of the elbow. A second independent therapist held the participant’s arm in this position, or alternatively, the participant’s arm was supported by a chair or pillow. If this position was not tolerated, the participant’s elbow was flexed to a point where the symptoms were diminished. With keeping this position, the treating therapist cradled the patient’s neck and performed oscillatory lateral glide mobilization towards the non-symptomatic side at grade 3 [29] for 1 min and 3 sets [30].

### Comparison group

The participant was in a prone position. Then, the treating therapist applied a minimal superficial circular pressure on the skin at the most symptomatic level of the cervical vertebra (central or unilateral) for 2 min and 3 sets.

### Both experimental group and comparison group

Strengthening exercises to the deep neck flexor muscles were prescribed for both groups. The participant was in a supine position with the neck in a neutral position. The participant was asked to straighten the curve of the neck by nodding the head for 10 s for 10 repetitions.

The participants were informed that the manual therapy techniques and exercise are safe and not harmful. However, these techniques may cause some discomfort for few minutes after the treatment, which is usually normal.

### Outcome measures

All the following outcome measurements were assessed by the assessor at baseline, 5 min after the first session, and 5 min after the sixth session, except for the neck disability index (NDI) which was only conducted at baseline and after the sixth session.

### Primary outcomes

#### Pressure pain threshold (PPT)

PPT was measured using an electronic algometer (Somedic AB, Sösdala, Sweden) with a probe of 1 cm^2^ diameter. The intra-rater reliability of pressure algometer was satisfactory or good (*r*=0.78–0.93) at different areas on the neck and shoulder [31]. In patients with neck pain, the PPT has a minimal detectable change (MDC) value of 5.7–8.7 N/cm^2^ (equivalent to 57–87 kPa) for the neck [32] and 171.3 kPa for the tibialis anterior [33]. The algometer was applied perpendicular to four areas on the body (ipsilateral side): on the most painful level of the cervical spine (facet joint), on the C7 hand dermatome (palmar of the 2nd metacarpal), on the affected dermatome (i.e., C5, C6, or C8), and on the bulk of the tibialis anterior muscle ~2.5 cm lateral and 5 cm inferior to the tibial tuberosity to assess the presence of widespread sensitivity. Pressure was applied at a rate of 40 kPa/s and the participants were asked to press a switch when the pressure sensation started to become painful. The test was repeated thrice at each site within 15-s intervals between measurements, and the average was used for analysis [34].

#### Heat/cold pain thresholds (HPT/CPT)

HPT and CPT were measured using a SenseLab Thermotest System (Somedic AB, Sösdala, Sweden). The validity and reliability of thermal pain thresholds are well established [35, 36]. The thermode was applied directly at four areas on the body (ipsilateral side): the most painful level of the cervical spine, the C7 hand dermatome (dorsum of the 2nd metacarpal), the affected dermatome (i.e., C5, C6, or C8) [20], and the belly of the tibialis anterior muscle. From a baseline of 32°C, the temperature was either increased to a maximum cut-off temperature of 52°C or decreased to a minimum cut-off temperature of 5°C at a rate of 1°C per second in each direction to prevent thermal injury. Participants were asked to press a switch when they felt heat or cold pain (painful threshold). If the pain threshold was not reached before the cut-off temperatures, the value was recorded as 52°C or 5°C for that trial. Three repetitions were performed and the interstimulus interval was at least 10 s for pain thresholds, and the average was used for analysis [34].

### Secondary outcomes

#### Numeric Pain Rating Scale (NPRS)

NPRS was used to assess the current intensity of pain. The NPRS has construct validity, acceptable responsiveness, and test-retest reliability [37]. The NPRS has a minimal clinically important difference (MCID) of 2.2 points in patients with neck pain with upper limb symptoms and 1.5 points in patients without upper limb symptoms [37]. This scale ranges from 0 (“no pain”) to 10 (“worst pain imaginable”).

#### The Neck Disability Index (NDI)

NDI was used to measure the patient level of disability. It comprises 10 items: 7 items pertinent to activities of daily living, 2 items pertinent to pain intensity, and 1 item pertinent to concentration. An item is scored from 0 to 5 with a total score raw score (0–50) or percentage score (0–100%). Raw score was used in this study. The higher the score, the higher the degree of neck disability. The NDI demonstrated acceptable responsiveness, construct validity, and fair test-retest reliability in patients with cervical radiculopathy [37]. The Arabic version of the NDI, which we used, is valid and reliable [38]. The MCID and MDC of the NDI in cervical radiculopathy is 8.5 and 13.4 points, respectively [37].

#### Active cervical range of motion (ROM)

Active cervical ROM was examined by using the cervical ROM device (Performance Attainment Associates, Roseville, USA). This device is valid and reliable to measure cervical ROM [39]. The participant sat on a chair with their hands rested on their thighs, and the device was placed around their head. The participant performed active neck movements in all directions until reaching full available range. If a movement was painful to a specific direction, the participant was asked to move to just the beginning of pain production. Each movement was recorded 3 times, and the average value was used for analysis [28]. The MDC values for the neck movements ranged from 3.6 to 6.5°, and the standard errors of measurement (SEM) ranged from 1.6 to 2.8° [39].

### Statistical analysis

All statistical analysis was performed by using IBM SPSS version 20 (IBM Corp, Armonk, USA). The baseline characteristics of patients in both groups were reported using the mean ± standard deviation for quantitative variables (age, body mass index (BMI), NPRS, and durations of treatment and symptoms) and frequency for qualitative variables (gender, affected side and affected dermatome, painful level, and number of medicines). The Shapiro-Wilk test was used separately for each group and for each outcome measure to test the normality of distribution. It revealed that all the measurements were normally distributed. Therefore, a mixed-model ANOVA was used to analyze the differences in the outcome measures between the groups (2 groups, 3-time levels). The Bonferroni corrections were applied to reduce the chance of type I error (false positive) associated with multiple comparisons [40]. To evaluate between-group differences in the dependent variables from baseline, independent *t* tests were used to compare changes in mean scores with 95% confidence intervals. Intention-to-treat was applied for 1 participant in the experimental group and 2 participants in the comparison group by replacing the missing data with the mean of the variables of the other group [41]. Pearson correlation coefficients were applied to examine the relationship between the self-report measures (NPRS and NDI) and QSTs (PPT, HPT, and CPT).

## Results

**Fig. 1:**
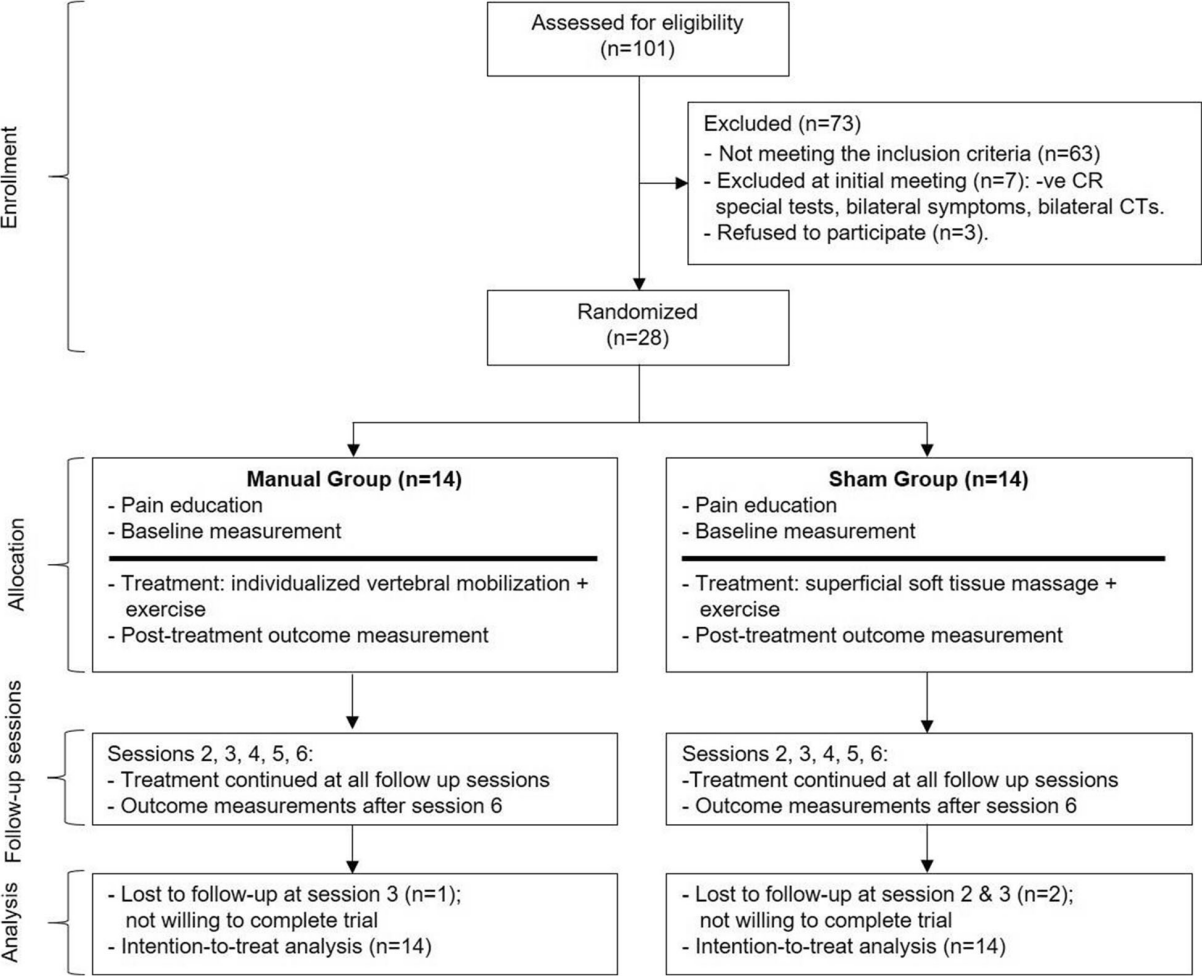
CONSORT flow diagram

Figure 1 summarizes the study recruitment and procedure. One hundred and one patients were screened for eligibility. Of these, 70 did not meet the inclusion criteria and 3 refused to continue in the study. This resulted in a total of 28 participants (25 females and 3 males) with a history of chronic cervical radiculopathy, who were randomized to the experimental group or the comparison group. One participant in the experimental group and two of the comparison group discontinued. None of the drop-outs, to our knowledge, was due to adverse events related to the treatments.

Baseline characteristics of the patients in both groups are presented in Table 1. The mean age of the patients was 42 years in both groups. This age is younger than the mean age that was previously reported (47.6–48.2 years) [42]. The mean duration of symptoms was 27±25 months in the experimental group and 22±29 months in the comparison group. The right side of the cervical spine was affected in 9 patients, and the left side was affected in 5 patients in both groups alike. The most involved dermatome level was C6 in both the experimental (7 patients) and comparison (5 patients) group. The most painful cervical levels were C4, C5, C6, and/or C7.

**Table 1:** Participants’ characteristics at baseline

Variable	Experimental group (***n***=14)	Comparison group (***n***=14)
Gender (female/male)	12/2	13/1
Age (years)	42±7	42±7
BMI (kg/m^2^)	32±6	29±5
NPRS (0–10)	6±1	6±2
NDI (0–50)	36±7	35±14
Treatment (weeks)	4±1	3±1
Duration of symptoms (months)	27±25	22±29
Affected side		
Left	5	5
Right	9	9
Dermatome involved		
C5	0	1
C5&C7	1	0
C6	7	5
C6&C7	2	2
C7	4	4
C8	0	2
Most painful level		
C4	2	1
C5	4	4
C6	5	4
C7	3	5
Medication^a^		
Yes	9	6
No	5	8

*Abbreviations*: *BMI* Body mass index, *NDI* Neck disability index, *NPRS* Numeric pain rating scale

Continuous values are expressed in mean ± standard deviation

^a^Included non-steroidal anti-inflammatory drugs, analgesics, and vitamin B complex

The means of the outcome measures at baseline are presented in Table 2. The difference in the outcome measures over the study period between the experimental group and the comparison group is shown in Table 3. Overall, there were improvements in the experimental group compared with the comparison group in the localized mechanical pressure hypersensitivity (PPT), self-report measures (NPRS, NDI), and cervical ROM; but no improvements in the thermal sensitivity (HPT and CPT) (see details below).

**Table 2:** Outcome measures in both groups at baseline

Outcome measure	Experimental group (***n***=14) (mean±SD)	Comparison group (***n***=14) (mean±SD)	Mean difference (95% confidence interval)
***PPT (kPa)***			
Cervical spine	347±214	294±100	53 (−77.1, 182.4)
C7 hand	476±184.1	435±130	41 (−82.4, 165.3)
Affected dermatome	421±128	424±187.7	−2 (−163.2, 157.9)
Tibialis anterior	519±173.6	475±148.1	44 (−81.2, 169.2)
***HPT (°C)***			
Cervical spine	45.2±4.2	45.6±4.4	−0.4 (−3.8, 2.9)
C7 hand	45.3±4	45.6±3.2	−0.3 (−2.5, 3.1)
Affected dermatome	45.5±3.2	44.9±2.2	0.7 (−1.9, 3.3)
Tibialis anterior	46.2±3.4	46.1±2.5	0.2 (−2.1, 2.5)
***CPT (°C)***			
Cervical spine	15.4±8.6	16.3±10	−0.9 (−8.1, 6.3)
C7 hand	17.1±9.1	18.8±7	−1.7 (−8.1, 4.6)
Affected dermatome	18.3±9.4	19.5±7.8	−1.2 (−9.6, 7.1)
Tibialis anterior	12.7±9.4	18.9±10.2	-6.2 (-13.8, 1.4)
***NPRS (0–10)***	6.4±1.3	6.4±1.7	0 (−1.2, 1.2)
***NDI (0–50)***	36.5±7.2	35.3±13.9	1.2 (−7.4, 9.7)
***Cervical ROM (degree)***			
Flexion	48±11.4	43±11.9	5 (−3.6, 14.5)
Extension	52±19.5	48±15.9	−4 (−17.7, 9.9)
Rotation (affected)	59±13.3	57±11.8	−2 (−7.0, 12.6)
Rotation (unaffected)	61±8.2	62±10.3	−1 (−8.7, 5.7)
Lateral flexion (affected)	33±8.7	32±8.1	−1 (−5.3, 7.8)
Lateral flexion (unaffected)	34±5.7	39±7.8	5 (−0.8, 9.8)

*Abbreviations*: *CPT* Cold pain threshold, *HPT* Heat pain threshold, *NDI* Neck disability index, *NPRS* Numeric pain rating scale, *ROM* Range of motion, *PPT* Pressure pain threshold, *SD* Standard deviation

**Table 3:** Mean change from baseline in within-group and in between-group for the outcome measures immediately and session 6 after intervention

Outcome measure	Within-group mean difference (95% CI)		Between-group mean difference (95% CI)
	Experimental group (***n***=14)	Comparison group (***n***=14)	
***PPT (kPa)***			
*Cervical spine*			
Immediately	35 (−4.6, 75.4)	−12 (−52.2, 27.8)	48 (−9.0, 104)
Session 6	124^a^ (57, 191.1)	67 (−.43, 133.7)	57 (−37.4, 152.2)
*C7 hand*			
Immediately	−2 (−58.9, 63.7)	13 (−48.4, 74.3)	−15 (−102.0, 71.5)
Session 6	99^a^ (3.6, 194.9)	83.6 (−12.1, 179.4)	16 (−119.7, 151.0)
*Affected dermatome*			
Immediately	−19 (−95.0, 56.9)	−30 (−106.3, 45.6)	11 (−96.1, 118.7)
Session 6	75 (−27.5, 177.4)	23 (−79.8, 125.1)	52 (−92.5, 197.2)
*Tibialis anterior*			
Immediately	−37 (−117.2, 44.1)	1 (−79.3, 81.9)	−38 (−152.0, 76.2)
Session 6	79 (−5.8, 162.9)	83 (−1.7, 166.9)	−4 (−123.4, 115.3)
***HPT (°C)***			
*Cervical spine*			
Immediately	0.6 (−1.5, 2.8)	−2.3 (−4.5, −0.2)	3.0 (−0.1, 6.0)
Session 6	0 (−2.0, 2.1)	−0.6 (−2.7, 1.4)	0.6 (−2.3, 3.5)
*C7 hand*			
Immediately	0.1 (1.0, 1.4)	−0.5 (−1.7, 0.7)	0.6 (−1.0, 2.3)
Session 6	0.1 (−2.1, 2.4)	−1.6 (−3.8, 0.7)	1.7 (−1.5, 4.8)
*Affected dermatome*			
Immediately	−1.1 (−3.0, 3.1)	0.0 (−1.8, 1.8)	−1.1 (−3.7, 1.6)
Session 6	−0.3 (−3.7, 3.1)	−1.1 (−4.3, 2.2)	0.8 (−3.9, 5.4)
*Tibialis anterior*			
Immediately	0.4 (−0.7, 1.4)	−0.8 (−1.9, 0.2)	1.2 (−0.3, 2.7)
Session 6	0.5 (−1.0, 1.9)	−0.1 (−1.7, 1.3)	0.6 (−1.5, 2.7)
***CPT (°C)***			
*Cervical spine*			
Immediately	−1.8 (−6.8, 3.2)	−3.4 (−8.4, 1.6)	−1.6 (−8.7, 5.4)
Session 6	1.7 (−3.6, 6.9)	−2.9 (−8.2, 2.3)	−4.7 (−12.1, 2.8)
*C7 hand*			
Immediately	−3.5 (−6.8, −0.4)	−1.3 (−4.4, 2.0)	2.4 (−2.2, 6.9)
Session 6	−0.9 (−5.4, 3.5)	−2.2 (−6.6, 2.3)	−1.2 (−7.4, 5.1)
*Affected dermatome*			
Immediately	−1.3 (−6.5, 3.7)	−2.3 (−7.2, 2.5)	−0.9 (−8.0, 6.1)
Session 6	0.6 (−4.5, 5.5)	−2.4 (−7.2, 2.3)	−3.0 (−9.9, 4.0)
*Tibialis anterior*			
Immediately	−3.2 (−5.9, −0.3)	0.2 (−2.5, 3.0)	3.3 (−0.6, 7.3)
Session 6	−2.2 (−5.9, 1.6)	1.7 (2.0, 5.5)	3.9 (−1.4, 9.1)
***NPRS (0-10)***			
Immediately	1.8^a^ (0.8, 2.8)	0.4 (−0.6, 1.3)	−1.4^a^ (−2.8, −0.0)
Session 6	3.9^a^ (2.4, 5.2)	1.2 (−0.2, 2.6)	−2.6^a^ (−4.6, −0.7)
***NDI (0-50)***			
Immediately	---	---	---
Session 6	16.2^a^ (9.4, 23.0)	2.4 (−4.3, 9.1)	−14.0^a^ (−23.3, −4.3)
***Cervical ROM (degree)***			
*Flexion*			
Immediately	4 (−3.4, 10.1)	4 (−2.6, 11.0)	−0.8 (−10.4, 8.8)
Session 6	5 (−2.1, 11.5)	4 (−2.4, 11.2)	0.3 (−9.3, 10.0)
*Extension*			
Immediately	0 (−5.8, 6.1)	−3 (−9.2, 2.7)	3.1 (−5.3, 11.5)
Session 6	10^a^ (1.3, 17.7)	−4 (−12.6, 3.8)	14.0^a^ (2.3, 25.5)
*Rotation (affected)*			
Immediately	6^a^ (0.6, 12.1)	−2 (−8.1, 3.4)	8.7^a^ (0.6, 16.8)
Session 6	12^a^ (7.7, 17.4)	−3 (−7.9, 1.8)	15.6^a^ (8.8, 22.5)
*Rotation (unaffected)*			
Immediately	3 (−3.4, 56.9)	0 (−5.6, 5.9)	2.1 (−6.0, 10.2)
Session 6	4 (−1.7, 8.3)	1 (−4.2, 5.9)	2.4 (−4.7, 9.5)
*Lateral flexion (affected)*			
Immediately	7^a^ (1.6, 11.4)	0 (−4.8, 5.0)	6.5 (−0.5, 13.4)
Session 6	8^a^ (3.2, 13.4)	−1 (−1.3, 13.4)	9.5^a^ (2.3, 16.8)
*Lateral flexion (unaffected)*			
Immediately	−1 (−4.6, 3.1)	0 (−4.1, 3.7)	−1.0 (−6.4, 4.5)
Session 6	0 (−4.2, 4.9)	−1 (−5.4, 3.5)	1.4 (−4.9, 7.7)

*Abbreviations*: *CI* Confidence interval, *CPT* Cold pain threshold, *HPT* Heat pain threshold, *NDI* Neck disability index, *NPRS* Numeric pain rating scale, *ROM* Range of motion, *PPT* Pressure pain threshold

^a^Indicates significant *P* value

For PPT, no group-by-time interaction was found at any of the areas tested: the cervical spine (*F*=1.999, *P*>0.050), the C7 hand dermatome (*F*=0.166, *P*> 0.050), the area of the affected dermatome (*F*=0.433, *P*>0.050), or the tibialis anterior (*F*=0.280, *P*>0.050). However, there was a time effect for PPT at the cervical spine (*F*=11.757, *P*<0.001), the C7 hand dermatome (*F*=7.314, *P*< 0.050), and the tibialis anterior (*F*=7.108, *P*< 0.050). There was no time effect at the area of the affected dermatome (*F*=3.194, *P*>0.050). PPT improved over the study period in the experimental group at the cervical spine (mean difference 124 kPa, 95% CI 57, 191.1) and C7 hand dermatome (mean difference 99 kPa, 95% CI 3.6, 194.9). However, no differences were found between both groups at any of the tested areas.

Thermal pain sensitivity was examined by HPT and CPT. There was no group-by-time interaction for HPT (*F*=0.460 to 2.657, *P*≥0.080) or CPT (*F*= 0.496 to 1.852, *P*≥0.167). There was also no time effect for either HPT (*F*=0.292 to 0.865, *P*≥ 0.433) or CPT (*F*=0.743 to 1.835, *P*≥0.170). Over time, HPT did not improve at the cervical spine (0.6°C, 95% CI −2.3, 3.5), C7 hand dermatome (1.7°C, 95% CI −1.5, 4.8), affected dermatome (0.8°C, 95% CI −3.9, 5.4), or tibialis anterior (0.6°C, 95% CI −1.5, 2.7). Similarly, the CPT did not improve at the cervical spine (−4.7°C, 95% CI −12.1, 2.8), C7 hand dermatome (−1.2°C, 95% CI −7.4, 5.1), affected dermatome (−3.0°C, 95% CI −9.9, 4.0), or tibialis anterior (3.9°C, 95% CI −1.4, 9.1).

For pain as measured by NPRS, a group-by-time effect (*F*=5.583, *P*<0.050) was found. The mean difference of NPRS over the study period was 3.9 (95% CI 2.4, 5.2) in the experimental group and 1.2 (95% CI −0.2, 2.6) in the comparison group, indicating an improvement of 2.6 points (95% CI −4.6, −0.7; *P*=0.010) in the experimental group.

As for NDI, there was a group-by-time effect (*F*=8.895, *P*<0.050). The mean difference of NDI was 16.2 (95% CI 9.4, 23.0) in the experimental group and 2.4 (95% CI −4.3, 9.1) in the comparison group, indicating an improvement of 14.0 points (95% CI −23.3, −4.3; *P*=0.006) in the experimental group.

For active cervical ROM, there was a group-by-time interaction for extension (*F*=4.819, *P*<0.050), rotation to the affected side (*F*=8.303, *P*<0.001), and lateral bending to the affected side (*F*=4.332, *P*<0.050). No group-by-time interaction was found for flexion (*F*=0.034, *P*=0.967), rotation to the unaffected side (*F*=0.240, *P*=0.788), and lateral bending to the unaffected side (*F*=0.301, *P*=0.742). There was an increase of 14.0° extension (95% CI 2.3, 25.5; *P*=0.021), 15.6° rotation to the affected side (95% CI 8.8, 22.5; *P*<0.001), and 9.5° lateral bending to the affected side (95% CI 2.3, 16.8; *P*=0.012) compared to the comparison group.

There were no correlations between the self-report measures (NPRS and NDI) and QST’s (PPT, HPT, and CPT). A strong positive correlation (*r* = 0.80, *P* < 0.001) was found between NPRS and NDI, indicating that improvement in pain intensity is associated with improvement in in neck function (Table 4).

**Table 4:** Pearson correlation coefficient for the relationship between the outcome measures

Outcomes	***r***^**2**^	***P*** value
**NPRS** vs.		
NDI	0.82	< 0.000
CROM—extension	−0.02	0.918
CROM—lateral flexion (affected)	−0.05	0.817
CROM—rotation (affected)	−0.32	0.100
PPT—cervical spine	−0.33	0.086
PPT—affected dermatome	−0.03	0.906
HPT—cervical spine	0.04	0.833
HPT—affected dermatome	−0.17	0.475
CPT—cervical spine	0.11	0.587
CPT—affected dermatome	0.10	0.683
**NDI** vs.		
CROM—extension	−0.2	0.304
CROM—lateral flexion (affected)	−0.19	0.339
CROM—rotation (affected)	−0.26	0.18
PPT—cervical spine	−0.16	0.426
PPT—affected dermatome	0.16	0.538
HPT—cervical spine	0.07	0.727
HPT—affected dermatome	−0.22	0.362
CPT—cervical spine	−0.03	0.875
CPT—affected dermatome	0.22	0.37

*Abbreviations*: *NPRS* Numeric pain rating scale, *NDI* Neck disability index, *CROM* Cervical range of motion, *PPT* Pressure pain threshold, *HPT* Heat pain threshold, *CPT* Cold pain threshold

## Discussion

This is the first study that has investigated the short-term effects of manual therapy with exercise on the sensory features in patients with chronic cervical radiculopathy. The manual technique that was used in the experimental group was cervical vertebral mobilization, whereas the technique used in the comparison group was minimal superficial circular pressure on the skin. The results suggested that applying cervical vertebral mobilization for these patients yielded improvements in the local mechanical pressure hypersensitivity as well as self-report measures on pain intensity and neck function and active cervical ROM. No improvements were found in the thermal pain thresholds.

Reduction of mechanical pain was observed in the neck following cervical vertebral mobilization as demonstrated by increased PPT after session 6. The mean improvement of PPT on the neck in the experimental group after session 6 was 124 kPa, which exceeded the MDC of 87 kPa [32]. Sterling et al. [43] and Lopez-Lopez et al. [22] found a similar improvement in PPT at the neck in whiplash and chronic neck pain patients. Interestingly, PPT improved at the C7 dermatome at the hand (99 kPa) following cervical vertebral mobilization after session 6, which reached the MDC. Seven out of 14 (50%) participants in the experimental group demonstrated affected C7 dermatome. Previous studies showed that the most affected level in patients with cervical radiculopathy was the C7 nerve root [44]. We found no improvements in PPT at the area of the supposed affected dermatome. This may partially be attributed to the fact that the observed pattern of pain and numbness differed from the standard dermatomal pattern (“Netter diagram” distribution) in 46% of patients with cervical radiculopathy in a previous study [45].

Cold hyperalgesia is a feature of chronic cervical radiculopathy [7–9]. In our study, CPT did not change after cervical vertebral mobilization. However, the values of CPT in both groups at baseline at the neck (15.4–16.3 C°) and the hand (17.1–18 C°) were higher than the values of other studies on the same tested areas [7–9]. This may suggest that our patients already had cold hyperalgesia at baseline. In addition, the values of HPT at baseline at the hand and affected dermatome in our study are similar to the values in other studies [7, 8]. Other studies demonstrated that HPT/CPT did not change after vertebral mobilization in patients with different musculoskeletal conditions, such as lateral epicondylalgia [46] and chronic whiplash associated disorders [30].

The findings of NPRS corroborated those of PPT on the neck. Cervical vertebral mobilization resulted in pain reduction of 3.9 points as measured by the NPRS over the study period, more than the MCID of 2.2 points [37]. Our results are similar to previous studies on patients with cervical radiculopathy which used manual therapy with or without other techniques [16, 23, 27].

In this study, disability related to neck pain as measured by the NDI decreased after cervical vertebral mobilization (mean difference 16.2 points). This improvement was more than the MCID of 8.5 points or the MDC of 13.4 points [37]. Our findings are similar to the findings of other studies on cervical radiculopathy that reported a change of 17.8–22.4 points [16, 23].

Cervical ROM improved with cervical vertebral mobilization particularly after session 6 in extension (mean difference 14°), rotation to the affected side (~16°), and lateral bending to the affected side (~10°). This improvement was more than the values of MDC (5°, 4.9–6.1°, and 3.6–4.2°, respectively) [39]. Interestingly, these movements demonstrated to decrease the size of the intervertebral foramen [47], which in turn may compromise the nerve root. Improvement of ROM to these movements may be due to reduced swelling and edema of the nerve root as a result of vertebral mobilization and decrease in pain, allowing more ROM towards the narrowed intervertebral foramen. A previous study showed improvements in extension and bilateral lateral bending after 4 and 8 weeks of treatment [16].

The current study did not find relationships between the self-report measures (NPRS and NDI) and QSTs (PPT, HPT, and CPT). A systematic review and meta-analysis found no meaningful correlations between QSTs and pain or disability in patients with spinal pain [48]. Conversely, a more recent systematic review showed that QST can predict worse outcomes of pain and disability in musculoskeletal disorders [49]. However, this correlation was weak for both pain (*r* = 0.31, 95%CI 0.23 to 0.38) and disability (*r* = 0.30, 95%CI 0.19 to 0.40) [49]. An explanation for this weak correlation may be due to that both methods (self-report measures and QSTs) measure different constructs that are not directly related. Thus, QSTs may be a poor marker of central or peripheral sensitization, which may not play a key role in participants’ reporting of pain or disability [48]. Although QSTs may be useful in classifying individuals with chronic pain based on mechanisms, future research is required to further improve the clinical utility of QSTs [50].

### Study limitations

A limitation of this study is that we studied the effect of vertebral mobilization for a relatively short-term. Therefore, caution should be taken if a researcher or a clinician wants to apply these results in a longer follow-up. Another limitation is that this study did not include a “no treatment” group because the main aim was to modify only the vertebral mobilization techniques. However, we used minimal superficial pressure as a control technique. Previous studies also showed that manual therapy and exercises were more effective than “no treatment” [51]. The small number of patients in the current study might not allowed subgroup analyses. However, subgroup analysis is generally not recommended due to several issues such as emerging of false-positive result [52].

### Recommendations for practice

Localized mechanical pressure, but not thermal, hypersensitivity appears to be a clinical feature of the patients with chronic cervical radiculopathy. Cervical vertebral mobilization can be applied to reduce this mechanical hypersensitivity as well as to improve self-report pain, neck movement and function.

## Conclusion

The results of this study showed that in patients with chronic cervical radiculopathy vertebral mobilization of the cervical spine is effective in short-term improvement of pain intensity, neck function, and neck ROM as well as localized mechanical pressure hypersensitivity. On the other hand, there was no change in the thermal pain sensitivity. Further studies are needed to investigate the effects of cervical vertebral mobilization on somatosensory features for long-term in patients with cervical radiculopathy.

## Supplementary Information

**Additional file 1.** Consent form.

**Additional file 2.** Interventions.

## Data Availability

The datasets used and/or analyzed during this study are available from the corresponding author on reasonable request.

## Abbreviations

ANOVA: Analysis of variance
CI: Confidence interval
CONSORT: Consolidated Standards of Reporting Trials
CPT: Cold pain threshold
HPT: Heat pain threshold
IRB: Institutional Review Board
MCID: Minimal clinically important difference
MDC: Minimal detectable change
NDI: Neck disability index
NPRS: Numeric pain rating scale
QST: Quantitative sensory test
PPT: Pressure pain threshold
ROM: Range of motion
VAS: Visual analog scale
