# Supplementary material for: Effect of manual therapy with exercise in patients with chronic cervical radiculopathy: a randomized clinical trial
Source: Trials. 2021 Oct 18;22:716. doi: 10.1186/s13063-021-05690-y (PMC8525034; doi:10.1186/s13063-021-05690-y)
Supplement: Supplementary file 1 — Additional file 1. Consent form. [file 13063_2021_5690_MOESM1_ESM.docx]

**Consent form**

**TITLE:** Short-term Effect of Manual Therapy on Sensory Features in Patients with Cervical Radiculopathy: A randomized triple blind experimental study

**PRINCIPAL INVESTIGATOR:** Duaa Bamhair; BScPT; Master student (Department of Physical Therapy, University of Dammam).

**ADVISORS:** Ali Alshami; Associate Professor & Consultant of Physical Therapy PhD, MPT (clinical), GradCert (manipulative Therapy), GradCert (Clinical), BScPT, (Department of Physical Therapy, University of Dammam)

The main purpose of this research is to investigate the effect of manual therapy on sensory features in patients with cervical radiculopathy. Special measurements, tests and treatment will be used in this research.

You are being asked to participate in our study since you are complaining of neck pain radiating to the arm. If you participate in this study, you will be randomly allocated to in one of two treatment groups using manual therapy technique. The treatment will be for two sessions per week, for three weeks. The treatment session will take approximately 30 minutes except for the first and last sessions that will take approximately 90 minutes. The session will be undertaken in the Physical Therapy Department at King Abdul-Aziz Hospital and East Jeddah hospital in Jeddah.

**PROCEDURE OF EXAMINATION:**

We will do some special tests on your neck to confirm the diagnosis. At this stage, you will not continue with the study if the criteria of diagnosis do not fit with you. If you fit, the procedure below will follow. We will examine the neck range of motion by using a special device and we will locate the most painful area in the neck by hand palpation. After that, we will use special equipment's to measure your pain and sensation. At the time of treatment, you will rate your pain on a scale from 0 to 10 and answer some questions to fill out a scale on function before testing.

During the tests and intervention, you will be placed in different positions depending to the site to be tested and the type of treatment technique. You may feel a temporary discomfort with some tests. During the sensory testing, special devices will be used and instructions will be given to you to press a switch once the sensation of a pressure, temperature is becomes painful. The test will be applied on the following areas of the body: cervical spine, hand and upper part of leg. The tests will be repeated 3 times.

**PROCEDURE OF TREAMENT:**

1. **Manual therapy:**

The researcher will apply manual therapy technique on the symptomatic area of your neck for 2 minutes of 3 sets.

1. **Strengthening Exercise:**

You will be lying on the bed, and then the researcher will instruct you to flatten your neck by nodding your head and holding this position for 10 seconds and repeating it 10 times.

These techniques and exercise are safe and not harmful, you may feel some discomfort for few minutes after the treatment and that is usually normal. You may have a benefit from this study by improving your symptoms. The findings may help to develop more effective therapy techniques for people with radiculopathy in the future.

Your participation in this study will be voluntary and you have the right to withdraw from the study at any time, without affecting the ongoing treatment of your condition in any way. Your privacy while participating in the study will be maintained all the time. All your data either electronic or printed will be kept in a personal locker in the Department of Physical Therapy at the hospital. The results of this study will be published in related scientific journals as a part of a master research project in related scientific journals, but your identity will not be disclosed at all. Feedback on the treatment results will be provided on request and a summary of the overall outcomes of the study will be available at the completion of the research project.

If you have any questions about your participation in this study or in case of any complications, you can contact the researcher on the following email: [duaa_bamhair@hotmail.com](mailto:duaa_bamhair@hotmail.com) or Mobile: 0500613675.

**I have read and understood the details above and have had the opportunity to ask questions. I understand that my participation is voluntary and that I have the right to withdraw at any time. I agree to participate in this study.**

Signed: (participant)_______________________________ Date: _______________

Signed: (investigator)______________________________ Date: _______________
