# Supplementary material for: Effect of manual therapy with exercise in patients with chronic cervical radiculopathy: a randomized clinical trial
Source: Trials. 2021 Oct 18;22:716. doi: 10.1186/s13063-021-05690-y (PMC8525034; doi:10.1186/s13063-021-05690-y)

**Effect of manual therapy with exercise in patients with chronic cervical radiculopathy: A randomized clinical trial**

**Supplement file: Interventions**

Before the first session started, all participants in both groups were provided with a standardized verbal education about pain based on a previous protocol. This education includes the following instructions/information:

1. "No brain, no pain". This means that pain is often present without tissue damage, that pain is often disproportionate to tissue damage, and that tissue damage (and nociception) does not, in itself, result in the feeling of pain.”
2. Decrease stress
3. Encourage physical activity
4. Do home exercise program (HEP).

Each participant had 6 treatment sessions over 3-5 weeks, similar to a previous protocol.

**Experimental group**

The experimental group received an individualized cervical mobilization technique and exercise. The following cervical vertebral mobilization technique was used based on the participants’ responses (i.e., reduction and/or centralization of symptoms): posterior-anterior (PA) or lateral vertebral glides.

For the PA vertebral glides, the participant was in a prone position and the treating therapist stood at the side of the participant’s head. The tips of the thumbs were placed in opposition at the level of the spinous process (for central PA) or at the level of the facet joint (for unilateral PA) of the cervical vertebra. Then, the treating therapist applied an oscillatory pressure of grade three on the most painful level for 2 minutes and 3 sets (Figure 1).


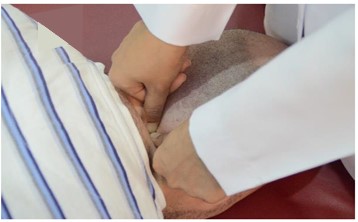


Figure 1. Cervical manual therapy techniques: Postero-anterior mobilization glide on right facet joint.

For the cervical lateral vertebral glides, the participant was supine, and the treating therapist placed the symptomatic patient's upper limb into an upper limb neurodynamic test 1 (median nerve bias) position as tolerated: abduction of the shoulder, lateral rotation of the shoulder, supination of the forearm, extension of the wrist and finger, and extension of the elbow. A second independent therapist held the participant's arm in this position, or alternatively, the participant's arm was supported by a chair or pillow. If this position was not tolerated, the participant's elbow was flexed to a point where the symptoms were diminished. With keeping this position, the treating therapist cradled the patient's neck and performed oscillatory lateral glide mobilization towards the non-symptomatic side at grade 3 for 1 minute and 3 sets (Figure 2).


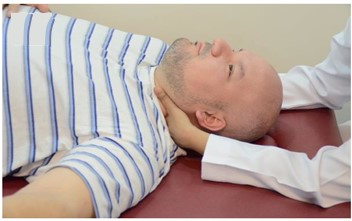


Figure 2. Cervical manual therapy techniques: cervical lateral vertebral glide.

**Comparison group**

The comparison group received minimal superficial pressure on the skin and exercise. The participant was in a prone position. Then, the treating therapist applied a minimal superficial circular pressure on the skin at the most symptomatic level of the cervical vertebra (central or unilateral) for 2 minutes and 3 sets.


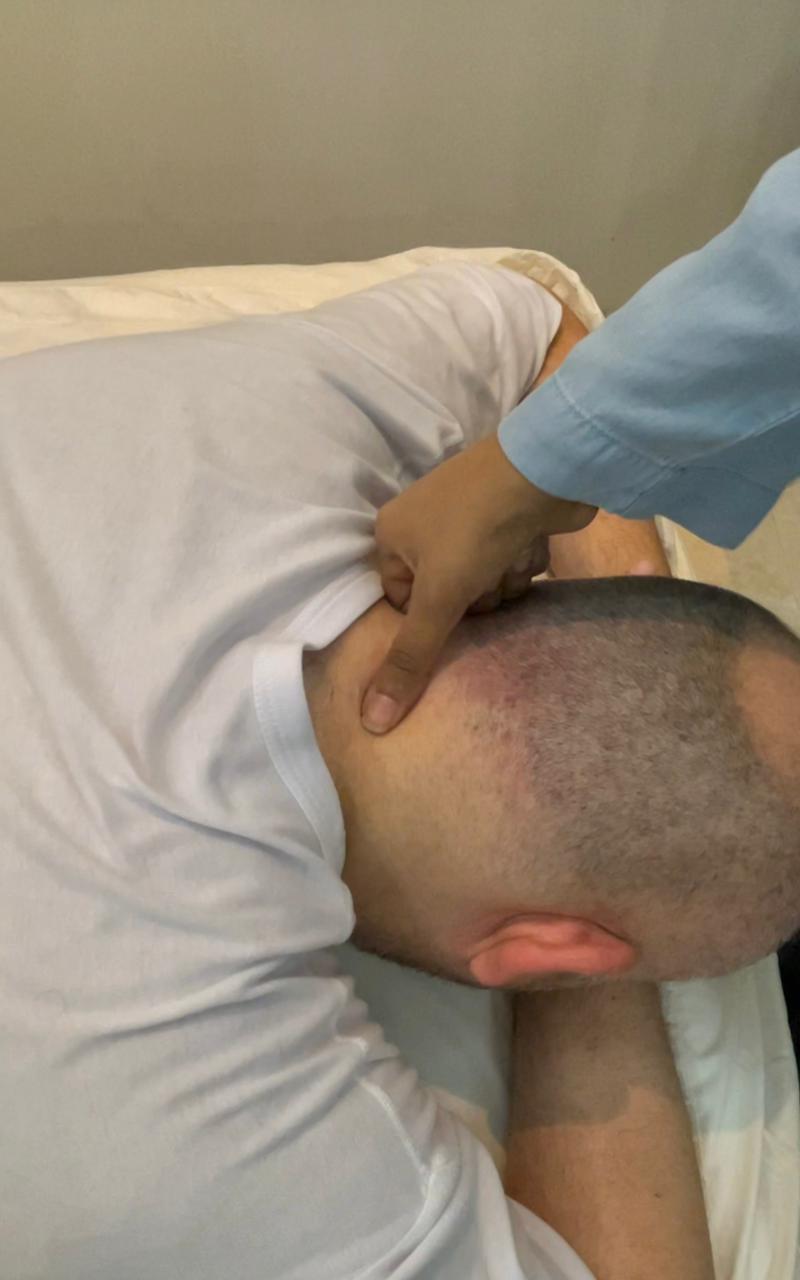


Figure 3. minimal superficial circular pressure on the skin at the most symptomatic level.

**Both experimental group and comparison group**

Strengthening exercises to the deep neck flexor muscles were prescribed for both groups. The participant was in a supine position with the neck in a neutral position. The participant was asked to straighten the curve of the neck by nodding the head for 10 seconds for 10 repetitions. This exercise was done once every day during the study period.

Figure 4. Deep neck flexor strengthening exercise: (A) starting position, (B) end position.


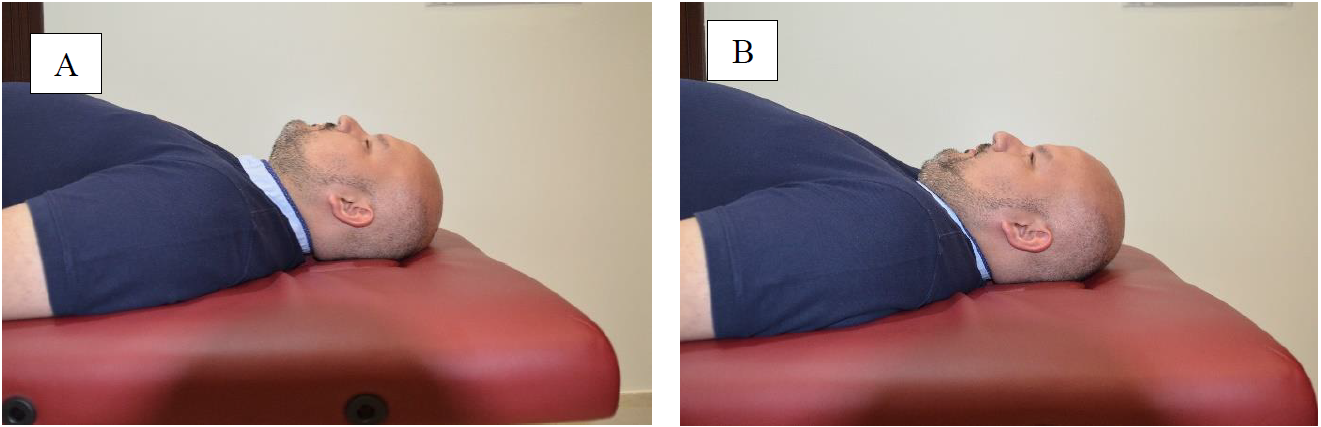

Supplement: Supplementary file 2 — Additional file 2. Interventions. [file 13063_2021_5690_MOESM2_ESM.docx]
